# The Book World of Medicine and Science

**Published:** 1899-04-15

**Authors:** 


### The Book World of Medicine and Science.

A Code of Rules for the Prevention of Infectious and Contagious Diseases in Schools. Issued by the Medical Officers of Schools Association. Fourth and Enlarged Edition. (London: J. and A. Churchill. 1899. Price 1s. 6d.)

It is eight years since the issue of the last edition of this work, and those responsible for it have decided that the time has arrived when its pronouncements may with advantage be revised. It had become clear that advancing knowledge had made certain alterations necessary, and that being so the occasion has been taken advantage of to revise the whole, with the result that, as the preface says, "almost every paragraph has been practically recast." Among other changes which have been made it will be noted that the "quarantine periods" allotted to scarlatina—oh, that unfortunate and deceptive word—and to small-pox have been reduced. On the other hand, attention is drawn to the fact that this quarantine of a person who has been exposed to infection is both uncertain and inadequate unless care be taken—by insisting on disinfection of the person and the clothing at the commencement of the quarantine—to ensure against his becoming infected by fomites during that period. In regard to nomenclature, it is to be observed that rubella and morbilli, the official names as they appear in "The Nomenclature of Diseases" are adopted as the synonyms of the diseases known as German measles and measles respectively. The term "epidemic roseola," however, which in the Nomenclature is associated with German measles is used in this work to represent "the common infectious exanthem which frequently bears a close resemblance to rubella, and for which the term epidemic roseola has been retained as a convenient synonym," a disease, however, which, as it is named in defiance of the official nomenclature issued by the Royal College of Physicians might well have received a more exact definition. Influenza is a disease the inclusion or exclusion of which in the list of those dealt with has, it is said, been a subject of careful consideration, but it has been decided that the ascertained facts in regard to it are not yet such as to admit of their being usefully summarised for precise and universal application. Tuberculosis, again, has not been included because, like typhoid fever, which also is not noticed in this work, "any attempt to do so could only result in directions either inadequate or superfluous." We hope that explanation will be found satisfying. Perhaps the most interesting pages of the book are those treating of the subject headed by the title "When free from infection." This period is made to be, in regard to measles, "not less than two weeks from the date of the appearance of the rash, convalescence being satisfactorily established"; in German measles, "not less than ten days from the date of the appearance of the rash;" in scarlet fever, however, it is "not less than six weeks from the date of the appearance of the rash, provided convalescence is completed and desquamation has ceased, and there is no sore throat, discharge from the ear, suppurating glands, or eczematous patches." The following are the quarantine periods, dating from exposure to infection, but always on the supposition that disinfection has been carried out at the commencement of the quarantine: Chicken-pox, 20 days' quarantine; diphtheria, 12 days; German measles and epidemic roseola, 20 days; measles, 16 days; mumps, 24 days; scarlet fever, 10 days; small-pox, 16 days; whooping-cough, 21 days. Altogether, it is a most useful book, and, from being the authority accepted by the masters of the great public schools, it is an almost necessary reference book for medical men who have to deal with the children of the well-to-do. Its dicta may well, however, be applied to the management of school children of all degrees.

The Principles of Bacteriology. By Dr. Ferdinand Hueppe, Professor of Hygiene in the University of Prague. Authorised Translation from the German by Dr. E. O. Jordan, Assistant Professor of Bacteriology in the University of Chicago. (Chicago: The Open Court Publishing Company; and London: Kegan Paul, Trench, Trubner, and Co. 1899.)

The great interest of the book before us lies in this, that it deals mainly with principles and with the broad relations of bacteriological science. What strikes one from the beginning is that it is "good reading," and that it deals in a wide spirit with the very important subjects on which it treats. It must, however, be admitted that it is throughout of a very controversial character, and that it therefore loses much of the value which a general impartial review of the scientific bearing of the facts of bacteriology would have had to the student. In fact, it can hardly be regarded as a student's book, notwithstanding that in the earlier chapters there is a good deal that is elementary. It is, however, interesting enough, and is an able exposition of the views of the author. Its keynote is the influence of environment upon the microbe, as shown by its altered activities, the new products—poisonous, chromogenic, and pathogenic—which it may produce, and even the variation in its form which may be set up, by alteration of the conditions under which it is grown.

The following sentences, culled from the chapter on the causes of infectious disease, will perhaps best explain the views of the author on this subject: "The 'specific' bacteria are, therefore, not the true cause. That lies in the character of the nutrient medium; the bacteria can only elicit what is performed in the structure of the medium." Again, "Upon sifting all the available material I cannot find a fact which is in real harmony with Koch's conception of 'specific' disease germs. . . . I expressly acknowledge that we can distinguish species and genera among bacteria and other minute organisms. Such constancy as we observe, however, is not the mystical constancy of 'specific' essences, but a constancy made possible by the permanence of the environment. Micro-organisms change with the changes in their surroundings, and the placing of this fact on a sure footing constitutes the great advance that modern bacteriology has made beyond the standpoint reached by Koch."

The whole argument is put forward with considerable force, and the author's views as to the prevention of infectious disease will be received with sympathy by those who hesitate about accepting the bacteriologist as the final arbiter in all question of treatment and diagnosis. The book is certainly one well worth the reading; but it must be read in a more or less critical spirit.

